# CSRR-Based Microwave Sensor for Dielectric Materials Characterization Applied to Soil Water Content Determination

**DOI:** 10.3390/s20010255

**Published:** 2020-01-01

**Authors:** João G. D. Oliveira, Erica N. M. G. Pinto, Valdemir P. Silva Neto, Adaildo G. D’Assunção

**Affiliations:** 1 Department of Communication Engineering, Federal University of Rio Grande do Norte, Caixa Postal 1655, Natal CEP 59078-970, RN, Brazil; gjoao187@gmail.com (J.G.D.O.); vpraxedes.neto@gmail.com (V.P.S.N.); 2 Avenida Universitária Leto Fernandes, Federal Rural University of the Semi-Arid, Caraúbas CEP 59780-000, RN, Brazil; erica.gurgel@ufersa.edu.br

**Keywords:** soil water content, SWC, CSRR, microwave sensor, relative permittivity measurement, slotted circular patch antenna

## Abstract

A new and compact sensor based on the complementary split-ring resonator (CSRR) structure is proposed to characterize the relative permittivity of various dielectric materials, enabling the determination of soil water content (SWC). The proposed sensor consists of a circular microstrip patch antenna supporting a 3D-printed small cylindrical container made out of Acrylonitrile-Butadiene-Styrene (ABS) filament. The principle of operation is based on the shifting of two of the antenna resonant frequencies caused by changing the relative permittivity of the material under test (MUT). Simulations are performed enabling the development of an empirical model of analysis. The sensitivity of the sensor is investigated and its effectiveness is analyzed by characterizing typical dielectric materials. The proposed sensor, which can be applied to characterize different types of dielectric materials, is used to determine the percentage of water contained in different soil types. Prototypes are fabricated and measured and the obtained results are compared with results from other research works, to validate the proposed sensor effectiveness. Moreover, the sensor was used to determine the percentage of water concentration in quartz sand and red clay samples.

## 1. Introduction

Recent technological advances related to wireless and mobile communication technologies, increasingly demanding high transmission rates and low latency, have aroused the interest of researchers worldwide in the development of sensors that can get information on the electromagnetic characteristics of dielectric materials present in the communication channel or used in the manufacture of microwave devices and circuits.

Several analysis techniques have been developed but the most interesting are those with noninvasive and nondestructive characteristics with respect to the material under test (MUT). Planar sensors can be classified into several groups according to their application, principle of operation, or even the intrinsic characteristics of the used resonator. Among them, there is the group of planar sensors used in the characterization of microfluidics, which has been considered by several researchers [1,2,3,4,5]. Usually, substrate integrated waveguide (SIW) sensors are developed to characterize the complex permittivity of microfluidics [1,2] or are submerged in the liquid under test (LUT) [4,5].

Similarly, the recent development of sensors can be highlighted for several applications such as characterization of NaCL as solute in concentration in very dilute solutions [6,7,8], identification of the depth of burns in biological tissues [9], and characterization of chemical and organic materials [10,11].

Another group of sensors that has been widely investigated is that of sensors using typical resonator elements such as split-ring resonator (SRR) [12,13,14] or its complementary (CSRR), which consists of the negative image of the SRR [15,16,17,18,19,20]. Recently, SRR- and CSRR-based devices have attracted a great deal of attention from researchers due to their electrical characteristics, where in the resonance band the electric and magnetic fields appear concentrated nearby the ring opening, causing a subtle change in the electrical characteristics of the medium near this region to cause major disturbances in the resonance frequency of the element [17,18,19].

This work proposes a circular patch microstrip antenna based on the addition of a cut-out type CSRR in the resonant element, applied to the measurement of the relative permittivity of different materials (solid and sandy soils). The antenna sensitivity is verified by placing different MUT over the patch to shift its resonant frequencies at 2.26 GHz and 3.5 GHz. The proposed sensor has a high quality factor. In addition, an empirical model is proposed to obtain the relative permittivity results, based on the combination of the sensitivity results obtained for each of the two resonances. The performance of the sensor proposed in this work was validated by measuring the dielectric constant of three materials commonly used in literature—which are FR-4, Roger RO4003C, and glass—confirming the effectiveness of the proposed model. The proposed sensor is compared with another sensor presented in the literature [21] and the comparison results are in good agreement.

Moreover, the proposed sensor is used to characterize the water percentage in two different soil samples, which are quartz sand and red clay, taken from a coastal region in the Northeast of Brazil. In addition to the relative permittivity for different water concentrations, salinity measurements of the two soil samples were performed. The antenna sensor is simulated using Ansoft HFSS commercial software. Prototypes are fabricated and measured, and good agreement between simulation and measurement results is observed. The proposed sensor can be used in several types of measurement.

## 2. Materials and Methods

### 2.1. Sensor Design

**Figure 1:**
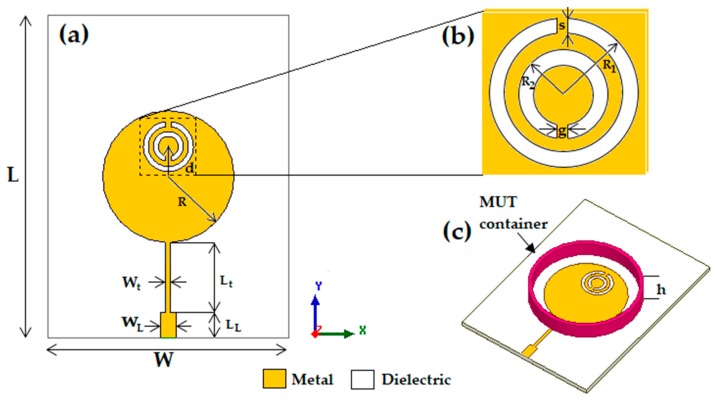
Proposed sensor: (**a**) Circular patch antenna; (**b**) double CSRR element; (**c**) sensor structure with the ABS 3D-printed container to hold the material under test (MUT).

The proposed sensor was initially modeled as a circular microstrip antenna patch antenna (Figure 1a), operating at 3.1 GHz, with a quarter-wavelength impedance matching circuit and a complementary split-ring resonator (CSRR) slotted element, as shown in Figure 1b, which caused a resonance frequency shift to 2.26 GHz and the emergence of a second resonance at 3.5 GHz. The choice of the microstrip antenna operating frequency at 3.1 GHz is related to the interest in developing a compact, low weight, low cost, and easy to manufacture narrowband antenna sensor to operate in the near field. In addition, the antenna sensor operating frequencies at 2.26 GHz and 3.5 GHz are in the same microwave range used in studies available in the literature [2,4].

Thereafter, a small cylindrical box made of Acrylonitrile-Butadiene-Styrene (ABS) TP20280 filament (Figure 1c) was made and placed over the patch to hold the MUT. The antenna dimensions are given in Table 1. The circular patch radius dimension was found using Equations (1) and (2), given in [22].

(1) $$a=\frac{F}{{\left\{1+\frac{2t}{{\pi \varepsilon }_{r}F}\left[\ln\left(\frac{\pi F}{2t}\right)+1.7726\right]\right\}}^{1/2}}$$

(2) $$F=\frac{8.791\times 10^{9}}{f_{r}\sqrt{\varepsilon_{r}}}$$

In (1), *a* is the patch antenna radius in cm, $f_{r}$ is the operating resonant frequency in GHz, F is given in cm, and the dielectric substrate parameters are thickness, *t*, in cm; relative permittivity, $\varepsilon_{r}$; and loss tangent, *tan*δ. In this work, a FR-4 substrate is used with *t* = 0.158 cm, $\varepsilon_{r}$ = 4.4, and *tan*δ = 0.02.

**Figure 2:**
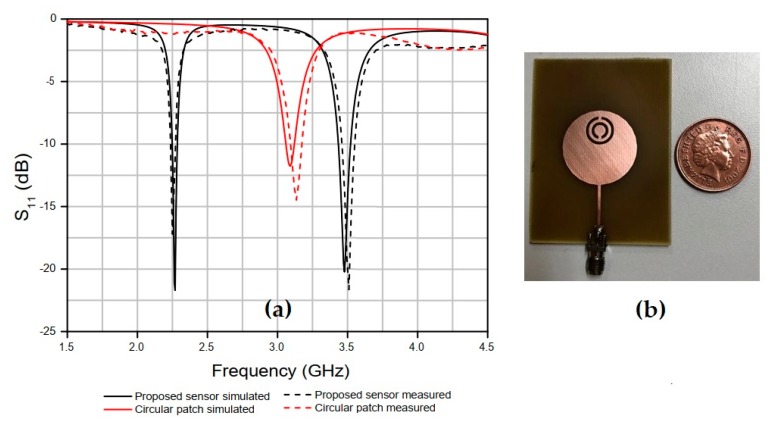
(**a**) Simulated and measured results of the reflection coefficient, S_11_ (dB), of the circular patch antenna and the proposed sensor with the double complementary split-ring resonator (CSRR) element; (**b**) photograph of the proposed sensor antenna.

The simulated and measured results of the reflection coefficient, S_11_ (dB), of the proposed circular patch antenna and sensor (with the slotted double CSRR element) are presented in Figure 2a, showing an 840 MHz decrease in the resonant frequency of the antenna and the occurrence of a second one, both related to the insertion of the CSRR element, resulting in a dual-band behavior.

The results shown in Figure 2 indicates that the proposed antenna has a high-quality factor, *Q*, which can be calculated using Equation (3) [23].

(3) $$Q=\frac{f_{0}}{BW_{3dB}}$$

where $f_{0}$ represents the resonant frequency and $BW_{3dB}$ describes the upper and lower frequencies bandwidth at 3 dB below peak. The value obtained for the quality factor of the first resonance was 105 and for the second one was 89. This parameter is commonly used in the characterization of the complex permittivity of materials [3,10]. Therefore, it was not used as one of the soil dielectric constant determination parameters, being used the analysis based on two resonance frequencies of the proposed sensor.

### 2.2. Principle of Operation and Sensitivity

In microwave resonators and at the resonant frequency, the energy of both electric and the magnetic field stored in the structure must be equal to each other. Thus, Equation (4), which relates the permeability and permittivity of the medium to the varying resonance frequency of the element, can be used to extract information from the properties of external materials that may cause these disturbances when interacting with the electromagnetic field [24].

(4) $$\frac{∆f_{r}}{f_{r}}=\frac{\int_{v}\left(∆\varepsilon E_{1}\cdot E_{0}+∆\mu H_{1}\cdot H_{0}\right)dv}{\int_{v}\left(\varepsilon_{0}{\left|E_{0}\right|}^{2}+\mu_{0}{\left|H_{0}\right|}^{2}\right)dv}$$

In (4), $∆f_{r}$ corresponds to the observed variation in the resonant frequency, $f_{r}$; $∆\varepsilon_{r}$ is the change in the relative permittivity; ∆μ is the change in the magnetic permeability; and $\varepsilon_{0}$ and $\mu_{0}$ are the permittivity and permeability of free space, respectively. Moreover, $E_{0}$ and $H_{0}$ are the electrical and magnetic fields distributions without external disturbances, respectivel; $E_{1}$ and $H_{1}$ are the corresponding electrical and magnetic fields distributions with external disturbances; and *v* represents the disturbed volume, which means the volume of the cavity that is in contact with the MUT. Similarly, the capacitance between the ends of the resonator element has a strong dependence on the medium permittivity and the induced current in the resonator element has a direct dependence on the medium permeability. Therefore, it can be said that CSRR elements have higher sensitivity to changes in the permittivity of the medium to which they are inserted [16], which justifies the application of CSRR in the proposed sensor geometry.

The proposed sensor was used to characterize samples of different materials which were separately inserted into the container placed over the patch of the microstrip antenna. The simulation of the sensor structure with the materials samples was performed using the Ansoft HFSS software.

**Figure 3:**
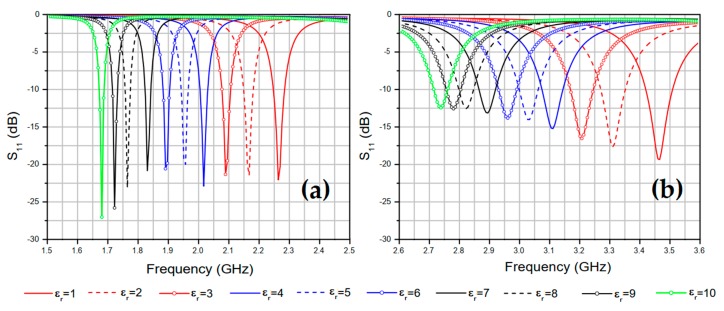
Reflection coefficient, S_11_ (dB), results for different (MUT) relative permittivity’s values at resonant frequencies (**a**) $fr_{1}$ and (**b**) $fr_{2}$.

In the simulation, the relative permittivity *ε_r_* of the MUT confined in the ABS filament container (Figure 1c) fabricated using a 3D printer was varied from 1 to 10 with a step of 1. The cylindrical container where the MUT is confined has an inner radius of 18 mm and a thickness of 1.2 mm. As the proposed antenna has two resonant frequencies ($fr_{1}$ and $fr_{2}$), the changes observed in the two resonance bands were analyzed separately enabling the development of an empirical model for the MUT characterization. The simulated results for the variation of the proposed antenna resonant frequencies, *f*_*r*1_ and *f*_*r*2_, as functions of the MUT relative permittivity are shown in Figure 3a,b, respectively. In the carried out simulation, dielectric losses are neglected.

Figure 3a,b show, respectively, that the resonance frequencies $fr_{1}$ and $fr_{2}$ of the proposed sensor have their values decreased as the relative permittivity value of the container-confined MUT sample is increased, as the total capacitance of the patch element also increases. The resonant frequency $fr_{1}$ changed from 2.26 GHz to 1.68 GHz, when the relative permittivity value $\varepsilon_{r}$ changed from 1 to 10, which represents a 25.8% reduction in the value of the lower resonant frequency of the proposed sensor. In Figure 3b, it is possible to notice that the resonance frequency $fr_{2}$ decreased from 3.46 GHz to 2.73 GHz when the relative permittivity of the confined MUT in the container changed from 1 to 10, which represents a 21.1% reduction in the higher resonant frequency of the proposed sensor.

The sensitivity evaluation of the proposed sensor (Figure 2b) was performed for each MUT by analyzing the variation observed in resonant frequency (∆*f_r_*), the percentage change in resonance frequency (PRFS), the enhancement of the percentage change in resonance frequency (PRFSE), sensitivity (S), and sensitivity enhancement (SE). Then, the results obtained were compared with those of another work carried out using a dual-band antenna sensor to characterize the relative permittivity of solid materials [21], according to Equations (5)–(9).

(5) $$∆f_{r}=f_{ru}-f_{rl}$$

(6) $$\mathrm{PRFS}=\frac{∆f_{r}}{f_{r}}=\frac{f_{ru}-f_{rl}}{f_{ru}}\times 100\%$$

(7) $$\mathrm{PRFSE}=\frac{{\mathrm{PRFS}}_{proposed}}{{\mathrm{PRFS}}_{reference}}$$

(8) $$S=\frac{∆f_{r}}{∆\varepsilon_{r}}=\frac{f_{ru}-f_{rl}}{\varepsilon_{ru}-\varepsilon_{rl}}$$

(9) $$\mathrm{SE}=\frac{S_{proposed}}{S_{reference}}$$

where $f_{ru}$ is the resonant frequency of the proposed antenna (or of the reference antenna shown in [21]) without MUT samples and $f_{rl}$ is the resonant frequency of the proposed antenna (or of the reference antenna) with MUT samples, $\varepsilon_{ru}$ is the relative permittivity of the medium when the antennas are without MUT samples, and $\varepsilon_{rl}$ is the relative permittivity of the medium when the antennas are without MUT samples. The parameter values shown in Figure 3a,b are summarized in Table 2 along with results presented in [21].

**Figure 4:**
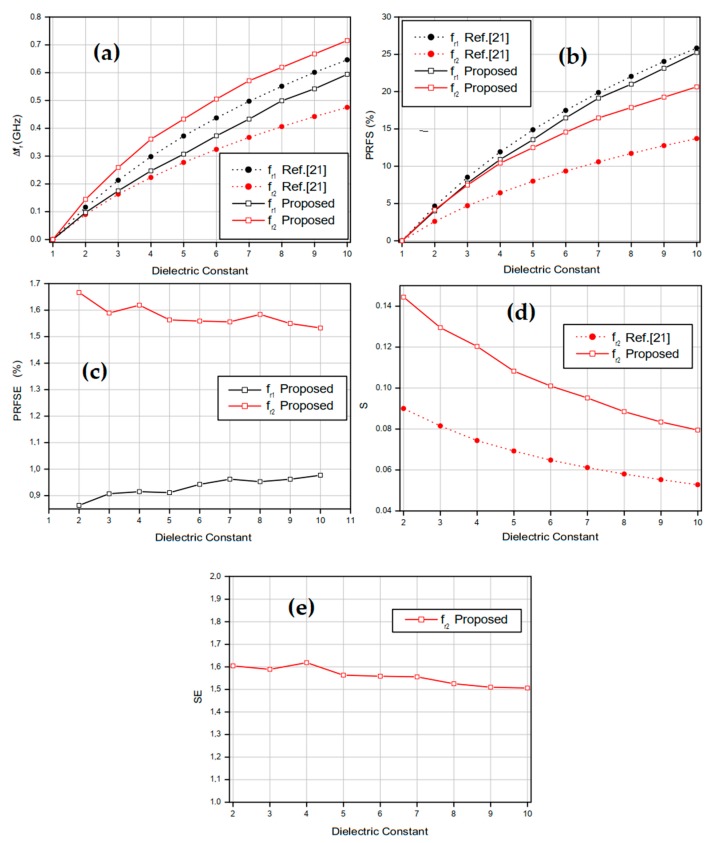
Sensitivity parameters results: (**a**) Resonant shift ($∆f_{r}$); (**b**) percentage relative frequency shift (PRFS); (**c**) percentage resonant frequency shift enhancement (PRFSE); (**d**) sensitivity (S); (**e**) sensitivity enhancement (SE).

Then, the simulated $f_{r}$ results shown in Table 2 were used in Equations (4)–(8) to determine the proposed sensor sensitivity parameters dependencies on the relative permittivity, as shown in Figure 4. In addition, the results of the dual-band sensor presented in [21] were included for comparison purpose. The shift in resonant frequency (∆*f_r_*) at $fr_{1}$ and $fr_{2}$ are presented in Figure 4a–c exhibit, respectively, the PRFS and PRFSE results at the sensor’s two resonance bands. Results of the sensors sensitivity (S) and sensitivity enhancement (SE) are shown in Figure 4d,e. In this case, only the sensitivity results at resonant frequency *f*_*r*2_ are presented because the *f*_*r*1_ value of the proposed sensor is different from that of the sensor developed in [21], not allowing a direct comparison.

According to Figure 4a,b, at the first resonant frequency $fr_{1}$, the resonance frequency shift $∆f_{r}$ and the percentage change in resonance frequency (PRFS) of the proposed sensor and of that presented in [21] are in good agreement. At the second resonant frequency $fr_{2}$, the proposed sensor-obtained results are much better than those of the sensor developed in [21]. In Figure 4a, when the MUT relative permittivity $\varepsilon_{r}$ is 8, the resonance frequency shift $∆f_{r}$ at $fr_{1}$ is 0.5 GHz for the proposed sensor and 0.551 GHz for the sensor presented in [21] while, at $fr_{2}$, when the MUT relative permittivity $\varepsilon_{r}$ is 5, the resonance frequency shift $∆f_{r}$ of the proposed sensor is 0.433 GHz and that of the sensor developed in [21] is 0.277 GHz.

In Figure 4c, for relative permittivity values greater than 8, the proposed sensor enhanced percentage change in resonance frequency (PRFSE) results at $fr_{1}$ are closer than those of the sensor presented in [21]. Additionally, when the relative permittivity value is 5, PRFSE = 0.91. At $fr_{2}$, PRFSE results are greater than 1.5 for the relative permittivity values in the range from 1 to 10. Figure 4d,e show that the sensitivity of the proposed sensor is higher for small values of the relative permittivity and exhibits a nonlinear dependence, as expected [19]. In addition, for a MUT sample with $\varepsilon_{r}=2$, the sensitivity S of the proposed sensor at the resonant frequency *fr*_2_ is 0.144 GHz, while the corresponding value of the sensor presented in [21] is 0.09 GHz. The enhanced sensitivity SE of the proposed sensor is 1.604.

## 3. Mathematical Model and Validation

### 3.1. Obtaining Relative Permittivity as a Function of Resonant Frequency

**Figure 5:**
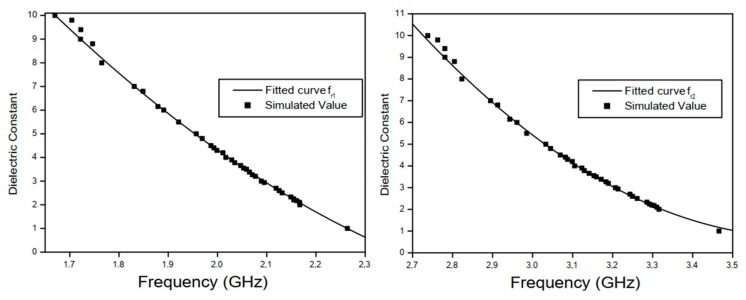
Fitted curves to describe the dependence of the proposed sensor resonant frequency with respect to different values of the permittivity value at (**a**) $fr_{1}\;\mathrm{and}$ (**b**) $fr_{2}$.

According to the results shown in Section 2, the variation of the MUT relative permittivity with the sensor resonance shift is characterized by a nonlinear behavior. The minimum values of the reflection coefficient (in dB) of the first and second resonance bands when the MUT relative permittivity is $\varepsilon_{MUT}=2$ are 2.167 GHz and 3.316 GHz, respectively. Similarly, when the MUT relative permittivity is $\varepsilon_{MUT}=8$, the first and second resonance bands of the proposed sensor occur at 1.765 GHz and 2.283 GHz, respectively. Thus, this frequency variation can be used to determine the MUT relative permittivity. As the proposed sensor has two resonance bands and each one has a different level of sensitivity with respect to the MUT relative permittivity, each case is analyzed separately, using curve fitting. The obtained curves used to fit each resonance band results are shown in Figure 5a,b, and the second-degree polynomials (expressions) are presented in Table 3.

The results shown in Figure 4 confirm that each of the proposed sensor resonances has different sensitivity levels when exposed to different electrical permittivity values. Thus, it is possible to analyze each one separately and use the results in a two-parameter analysis in order to make the final result more reliable.

Figure 5a,b show that the two resonance bands have different nonlinear behaviors for the variation of the MUT permittivity. Thus, the resonance bands polynomials expressions of *ε*_*r*1_ and *ε*_*r*2_ given in Table 3 are used to determine the polynomial expression for *ε_r_*, enabling the calculation of the final values of the MUT permittivity, along with the regression coefficient of determination, *R*^2^.

### 3.2. Proposed Sensor Validation

**Figure 6:**
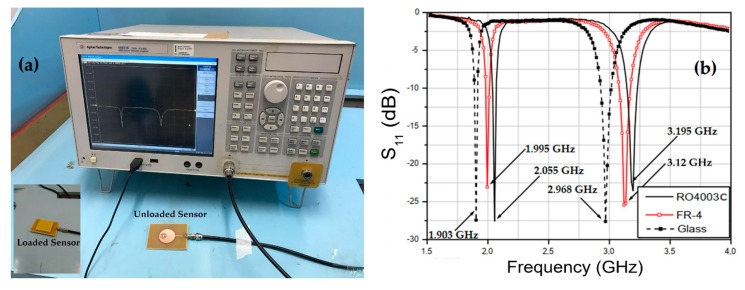
Experimental setup and results for the proposed sensor: (**a**) Measurement setup; (**b**) measured results for FR-4, glass, and Rogers RO4003C.

Then, the effectiveness of the proposed sensor was evaluated by measuring the relative permittivity of samples of various dielectric materials with results available in the literature for comparison purposes. Three dielectric materials commonly used as substrates in RF circuits—namely, FR-4 epoxy fiberglass, Rogers RO4003C, and glass—were analyzed. The used measurement setup is shown in Figure 6a, and the measured results are shown in Figure 6b. In the simulation, samples of dielectric materials with 4.8-mm height were analyzed. Thus, in the measurement characterization of FR-4 and Rogers RO4003C samples, multilayer geometries with 3 identical dielectric layers of 1.57 mm were used.

The results of the resonant frequencies shown in Figure 6b allow to calculate the relative permittivity of each MUT. Thus, for the FR-4 sample, the measured value of the first resonant frequency $fr_{1}$, which is 1.995 GHz, enabled us to calculate the relative permittivity value $\varepsilon_{r1}$ as 4.3835. Similarly, at the second resonance, the measured value for $fr_{2}$ which is 3.12 GHz enabled us to obtain the value of $\varepsilon_{r2}$ as 3.8969. Using the calculated values for $\varepsilon_{r1}$ and $\varepsilon_{r2}$, the relative permittivity of the FR-4 substrate is calculated as 4.3052 which, compared with the typical one of 4.4 (available in the literature), indicates a percentage error of 2.2%, validating the developed work.

The same procedure was applied to the results obtained in the measurement of glass and Roger RO4003C samples. The calculated values of these materials relative permittivity’s are $\varepsilon_{r}$(glass) = 5.8207 and $\varepsilon_{r}$(Roger RO4003C) = 3.4563. Usually, the glass-relative permittivity varies from 5 to 10, depending on the glass composition. Nevertheless, for microwave circuit applications, the typically used relative permittivity values of glass are 5.5 [25] and 6.2 [26]. For Rogers RO4003C laminates, the cited value of $\varepsilon_{r}$ is 3.55 [27]. Therefore, in the characterization of glass, the relative error is 3.08% (assuming $\varepsilon_{r}=6$, as reference value) and, in the case of the Rogers laminate, the error is 2.71%, showing agreement between usual and calculated values. The performance of the sensor proposed in this work was validated by measuring the dielectric constant of three materials commonly used in literature, which are FR-4, Rogers RO4003C, and glass, as shown in Figure 6. Agreement is observed between measured and typical results with relative differences lower than 3.08%.

## 4. Application: Soil Water Content (SWC) and Discussion

Recent technological advances are largely related to obtaining and using accurate information with a high degree of reliability. In some applications, this issue is of vital importance, as in the case of extracting information on soil moisture from the percentage of water in the sample. Some of them include, for example, monitoring of landslide and agricultural risk zones. Thus, several authors have proposed practical and simple ways to perform this type of characterization, such as a sensor that extracts moisture from soil samples based on Kopecky cylinders [28] or using high-frequency radar penetration [29]. The amount of water in the soil can be verified by measuring the relative permittivity of the sample [30], since the relative permittivity of the water is tens of times greater than the permittivity of the soil, with values of 80 for water and between 2 and 3 for dry sand.

Thus, a small interaction between the very different values of the relative permittivity of water and dry sand can cause significant changes in the characteristics of their mixture, indicating that the sensors used to characterize the relative permittivity of different dielectric materials can be used to characterize SWC and enabling the use of the sensor developed in this work in SWC applications.

In this work, measurements were made for two different soil types, namely, quartz sand and red clay. To better understand the difference between these soils, X-ray Fluorescence was performed. This is a multi-element technique used to obtain qualitative and quantitative information on the elemental composition of the samples, based on the production of characteristic X-ray emitted by the constituent elements of the soil. The equipment used was the Shimadzu EDX-720 model and the results of the analysis are shown in Table 4.

The soils used in this work were obtained in the coast region of Rio Grande do Norte. It is possible to observe from the chemical analysis that both materials are rich in Silica, Aluminum, and Iron. The sand has a larger number of elements because it was removed from the riverbed and may have been contaminated by pollutant residues present in the water. It is also a material with a larger grain size, causing water to stay on the grain surface but not causing particles to approach. The other soil used was red clay, which is composed of a larger amount of aluminum together with silica and has a very small grain size in relation to sand. This causes the grains to form clumps, trapping water within the sample, making the material plastic. The two soils studied here are characterized as sedimentary rocks and, due to erosion of our coastline, their presence is quite common. These soils can be used for the manufacture of cement, concrete, landfills, road infrastructure works, and agriculture.

**Figure 7:**
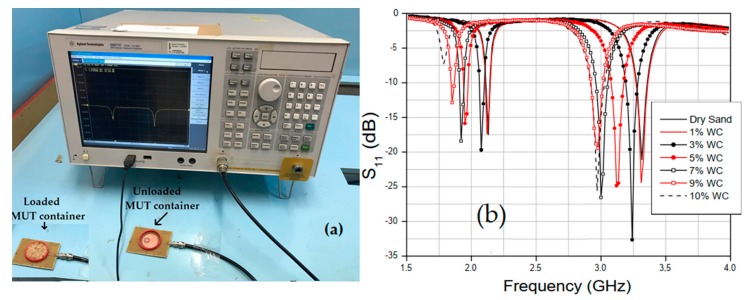
Quartz sand sample experimental procedure: (**a**) Measurement setup; (**b**) S_11_ variation for different Soil Water Content (SWC).

**Figure 8:**
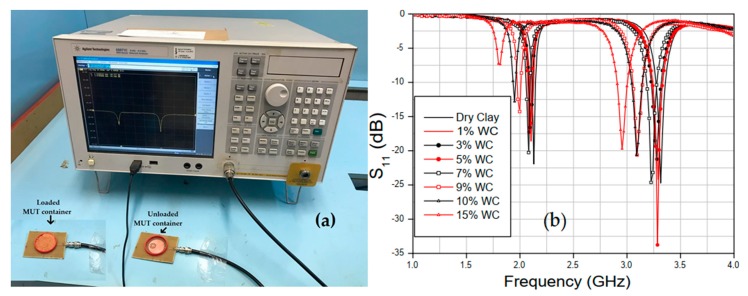
Red clay sample experimental procedure: (**a**) Measurement setup; (**b**) S_11_ variation for different SWC.

Measurements for 7 different water concentrations, with dry sand, 1%, 3%, 5%, 7%, 9%, and 10% of water and for red clay with 8 different concentrations with dry clay, with 1%, 3%, 5%, 7%, 9%, 10%, and 15% of water, the percentage calculation is given by (10) [31,32].

(10) $$SWC\left(\%\right)=\frac{m_{water}}{m_{DrySoil}}\times 100\%$$

where $m_{water}$ and $m_{DrySoil}$ is the weight (in gram) of the water and dry soils samples. The quartz sand setup is shown in Figure 7a, and the measured results of the sand reflection coefficient (S_11_, dB) for the three cases are shown in Figure 7b. The red clay setup is shown in Figure 8a and the results of S_11_ in Figure 8b for reproducibility. Measurements were performed in a climate-controlled room at 24 °C. The measured salinity values of the soil samples were 27.09 mg/L and 13.09 mg/L for quartz sand and red clay, respectively.

**Figure 9:**
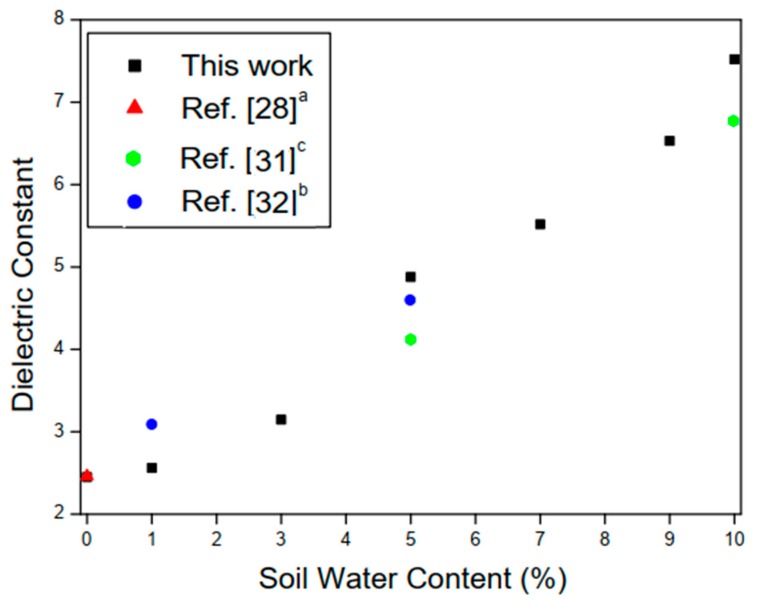
Measured results for different SWC (%) with quartz sand. ^a^ Soil sample obtained in the region of Banat in north-east of Serbia. ^b^ The values shown are the mean value of those obtained from measurements. ^c^ From Energy Dispersive X-ray (EDX) analysis, the sand soil sample is composed of 11.5% of Carbon, 28.1% Silicon, 0.1% Magnesium, 0.7% Iron, and 53% of Oxygen.

**Figure 10:**
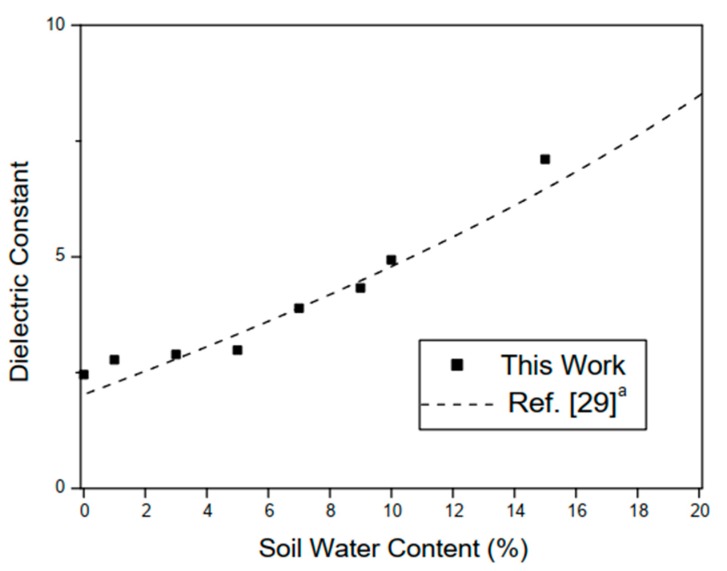
Measured results for different SWC (%) with red clay. ^a^ Soil obtained in the region of Yingtan, Jiangxi Province of China.

With the results obtained experimentally, it was possible to estimate the relative permittivity of the sand for different concentrations. It can be seen from Figure 8b that for small variations in water percentage, the sensor resonances had considerable shifts. For example, with 5% of water at $fr_{1}=1.95\;\mathrm{GHz}$ and at 10% at $fr_{1}=1.785\;\mathrm{GHz}$ a variation of 165 MHz is observed. The results of the relative permittivity of the two measured soils are compared with values obtained in the literature. For red clay, when a small amount of water was added, due to its absorption characteristic, the shift in the resonance frequency of the case between 0% and 7% of the amount of water was very small. The permittivity results obtained for the two different soil samples are shown in Figure 9 and Figure 10.

The experimental results presented in this work show good agreement with other authors’ reported works and the small differences in the results are due to the different characteristics of the soil samples, once the electrical characteristics of the soils are directly related to its chemical composition. Additionally, the compared soil samples (Figure 9 and Figure 10) have very different chemical characteristics and were extracted in different places.

The values of the soil dielectric constants for different water concentrations are shown in Table 5 and Table 6, for quartz sand and red clay, respectively.

For the quartz sand soil samples, measurements were made up to a concentration of 10% due to the fact that, with the concentration of 15% of water for the amount of material in the sensor, free water, i.e., not absorbed by the soil sample, drastically influenced the result obtained. The values measured for the red clay presented low relative permittivity values due to the absorption characteristic and high conductivity of this type of soil, thus causing higher dielectric losses [29].

**Figure 11:**
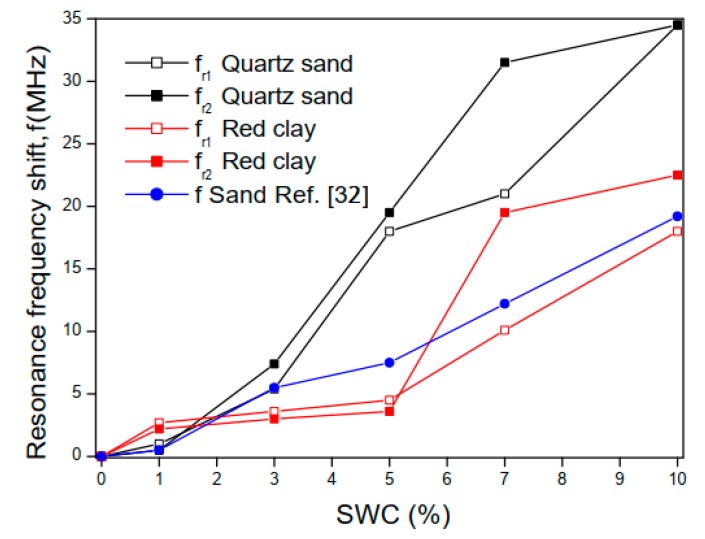
Measured results for the resonance frequency shift on quartz sand and red clay soil samples for different water concentrations.

The soil characterization was based on the sensitivity level of the proposed sensor, which is associated with shifts in resonant frequency. Measurements were performed on quartz sand and red clay soil samples for different water concentrations, as performed in [31]. Figure 11 shows a comparison between the results obtained in this work and those given in [31].

In Figure 11, it is possible to observe that for the quartz sand sample, the sensor proposed in this work has higher sensitivity levels than the one developed in [31], due to the higher resonance frequency shifts.

Table 7 presents a comparison of the key parameters of the sensor proposed in this work with other sensors reported in the literature.

## 5. Conclusions

A new and compact microwave sensor composed of a circular microstrip patch antenna with two slotted complementary split-ring resonators (CSRRs) was developed to characterize the relative permittivity of different dielectric materials and determine different water concentrations in different soil types. The operating principle is based on the difference between the resonant frequencies of the sensor with and without MUT samples. The sensitivity characteristics of the proposed sensor were analyzed and compared with the ones available in the literature, proving that the developed sensor has a great potential and can be used in several applications due to the required small amount of MUT sample, low cost, low weight, and ease of fabrication. In addition, an empirical model was proposed based on the behavior of the sensor at each resonance band, relating the resonant frequency to the permittivity of the materials under test (MUT). The sensor was used to characterize dielectric materials with known properties for comparison purpose and a good agreement between results was observed. It was also used to determine the percent water concentration in quartz sand and red clay samples. In addition, the sensor has great potential for use in medical, agricultural, and chemical applications due to the high sensitivity to small variations in the relative permittivity of different materials, low profile, small size, and planar structure.

The main contributions of this work are related to the modeling technique used to determine the dielectric constant based on the behavior of two resonances, the use of a sensor designed to determine the electrical permittivity of materials in the characterization of percentage soil water content (SWC), and the size reduction of the proposed sensor when compared to those presented in previous published works.

As a proposal, it would be interesting to investigate the combined use of CSRR and SRR elements to include the magnetic permeability characterization in the analysis, once ferromagnetic materials are present in many types of soils.

## Figures and Tables

**Table 1:** Proposed sensor structural parameters.

Parameter	Description	Value (mm)
*W*	Substrate Width	48
*L*	Substrate Length	64
*R*	Patch Radius	13.12
$W_{L}$	Microstrip Line Width	3
$L_{L}$	Microstrip Line Length	5
$W_{t}$	Quarter Wave Transformer Width	1
$L_{t}$	Quarter Wave Transformer Length	21
d	CSRR offset from source	6
$R_{1}$	CSRR External Radius	5
$R_{2}$	CSRR Internal Radius	3
s	CSRR Width	1
g	CSRR Air-gap	1
h	ABS recipient height	5

**Table 2:** Simulated $f_{r}$ (GHz) range values for changes in relative MUT permittivity.

Antenna	F	$\varepsilon_{r}$									
		1	2	3	4	5	6	7	8	9	10
Reference [21]	$fr_{1}$	2.5	2.384	2.287	2.202	2.128	2.063	2.003	1.949	1.899	1.854
Reference [21]	$fr_{2}$	3.466	3.376	3.303	3.243	3.189	3.142	3.099	3.06	3.024	2.991
Proposed	$fr_{1}$	2.264	2.173	2.089	2.017	1.957	1.891	1.831	1.788	1.740	1.692
Proposed	$fr_{2}$	3.466	3.321	3.207	3.105	3.033	2.961	2.895	2.846	2.798	2.750

**Table 3:** Fitting curves to obtain permittivity as function of resonance frequency.

Curve Fitting	$R^{2}$
$\varepsilon_{r1}=7.965fr_{1}^{2}-46.52fr_{1}+65.49$	0.9995
$\varepsilon_{r2}=10.31fr_{2}^{2}-75.79fr_{2}+140$	0.9994
$\varepsilon_{r}=0.8427\varepsilon_{r1}+0.1596\varepsilon_{r2}-0.01068$	0.9995

**Table 4:** X-ray fluorescence (XFR) analysis of two soils, values in (%).

Soil	Si	Al	Fe	k	Sr	Ca	Zr	Ti	Rb	Mn	S	Cr
**Sand**	50.106	8.774	12.584	7.738	5.538	4.733	1.988	1.176	1.041	0.179	0.133	-
**Red Clay**	46.585	23.725	18.178	1.001	-	-	5.038	3.463	-	-	0.509	0.206

**Table 5:** Measured results for quartz sand soil samples.

	WC (0%)	WC (1%)	WC (3%)	WC (5%)	WC (7%)	WC (9%)	WC (10%)
$fr_{1}\;\left(\mathrm{GHz}\right)$	2.130	2.120	2.076	1.950	1.920	1.851	1.785
$fr_{2}$ (GHz)	3.315	3.310	3.241	3.120	3.000	2.976	2.970
$\varepsilon_{r}$	2.450	2.560	3.146	4.877	5.517	6.530	7.520

**Table 6:** Measured results for red clay soil samples.

	WC (%)							
	0	1	3	5	7	9	10	15
$fr_{1}\;\left(\mathrm{GHz}\right)$	2.13	2.103	2.094	2.085	2.029	1.997	1.950	1.815
$fr_{2}$ (GHz)	3.315	3.293	3.279	3.285	3.120	3.095	3.090	2.955
$\varepsilon_{r}$	2.456	2.775	2.892	2.984	3.890	4.327	4.934	7.105

**Table 7:** Comparison of structural parameters with sensors reported in the literature.

Ref.	Structure	Area (mm^2^)	Sensing Parameter	Design Complexity	Char. Method	Two Resonances
[1]	Planar	N/A	$S_{11}$	Medium	Resonance	No
[2]	Planar	55×50	$S_{21}$	High	Resonance	No
[4]	Planar	40×35	$S_{21}$	Low	Resonance	No
[5]	Planar	N/A	$S_{21}$	Low	Resonance	No
[6]	Planar	55×63	$S_{21}$	Low	Transmission	N/A *
[7]	Planar	50×92	${S_{21}}^{DC}$ **	High	Transmission	No
[12]	Planar	80×40	$S_{21}$	Low	Resonance	Yes
[21]	Planar	80×80	$S_{11}$	Low	Resonance	Yes
[24]	Planar	N/A	$S_{21}$	Low	Resonance	No
This Work	Planar	48×64	$S_{11}$	Low	Resonance	Yes

* UWB sensor; ** Cross-mode transmission coefficient.

## References

[1] Silavwe E., Somjit N., Robertson I.D. (2016). A microfluidic-integrated SIW lab-on-substrate sensor for microliter liquid characterization. IEEE Sens. J..

[2] Wei Z., Huang J., Li J., Xu G., Ju Z., Liu X., Ni X. (2018). A High-sensitivity microfluidic sensor based on a substrate integrated waveguide re-entrant cavity for complex permittivity measurement of liquids. Sensors.

[3] Chretiennot T., Dubuc D., Grenier K. (2012). A microwave and microfluidic planar resonator for efficient and accurate complex permittivity characterization of aqueous solutions. IEEE Trans. Microw. Theory Tech..

[4] Reyes-Vera E., Acevedo-Osorio G., Arias-Correa M., Senior D.E. (2019). A submersible printed sensor based on a monopole-coupled split ring resonator for permittivity characterization. Sensors.

[5] Galindo-Romera G., Herraiz-Martínez F.J., Gil M., Martínez-Martínez J.J., Segovia-Vargas D. (2016). Submersible printed split-ring resonator-based sensor for thin-film detection and permittivity characterization. IEEE Sens. J..

[6] Soffiatti A., Max Y., Silva S.G., de Mendonça L.M. (2018). Microwave metamaterial-based sensor for dielectric characterization of liquids. Sensors.

[7] Vélez P., Muñoz-Enano J., Gil M., Mata-Contreras J., Martín F. (2019). Differential microfluidic sensors based on dumbbell-shaped defect ground structures in microstrip technology: Analysis, optimization, and applications. Sensors.

[8] Chahadih A., Cresson P.Y., Hamouda Z., Gu S., Mismer C., Lasri T. (2015). Microwave/microfluidic sensor fabricated on a flexible kapton substrate for complex permittivity characterization of liquids. Sens. Act. A Phys..

[9] Dinh T.H.N., Serfaty S., Joubert P.Y. (2019). Non-contact radiofrequency inductive sensor for the dielectric characterization of burn depth in organic tissues. Sensors.

[10] Helmy A.A., Kabiri S., Bajestan M.M., Entesari K. (2013). Complex permittivity detection of organic chemicals and mixtures using a 0.5–3-GHz miniaturized spectroscopy system. IEEE Trans. Microw. Theory Tech..

[11] Helmy A.A., Entesari K. (2012). A 1–8 GHz miniaturized spectroscopy system for permittivity detection and mixture characterization of organic chemicals. IEEE Trans. Microw. Theory Tech..

[12] KT M.S., Ansari M.A.H., Jha A.K., Akhtar M.J. (2017). Design of SRR-based microwave sensor for characterization of magnetodielectric substrates. IEEE Microw. Wirel. Compon. Lett..

[13] Shafi K.M., Jha A.K., Akhtar M.J. (2017). Improved planar resonant RF sensor for retrieval of permittivity and permeability of materials. IEEE Sens. J..

[14] Liu W., Sun H., Xu L. (2018). A Microwave method for dielectric characterization measurement of small liquids using a metamaterial-based sensor. Sensors.

[15] Haq T., Ruan C., Zhang X., Ullah S. (2019). Complementary metamaterial sensor for nondestructive evaluation of dielectric substrates. Sensors.

[16] Boybay M.S., Ramahi O.M. (2012). Material characterization using complementary split-ring resonators. IEEE Trans. Instrum. Meas..

[17] Vélez P., Su L., Grenier K., Mata-Contreras J., Dubuc D., Martín F. (2017). Microwave microfluidic sensor based on a microstrip splitter/combiner configuration and split ring resonators (SRRs) for dielectric characterization of liquids. IEEE Sens. J..

[18] Ebrahimi A., Withayachumnankul W., Al-Sarawi S., Abbott D. (2013). High-sensitivity metamaterial-inspired sensor for microfluidic dielectric characterization. IEEE Sens. J..

[19] Withayachumnankul W., Jaruwongrungsee K., Tuantranont A., Fumeaux C., Abbott D. (2013). Metamaterial-based microfluidic sensor for dielectric characterization. Sens. Act. A Phys..

[20] Yeo J., Lee J.I. (2019). High-sensitivity microwave sensor based on an interdigital-capacitor-shaped defected ground structure for permittivity characterization. Sensors.

[21] Yeo J., Lee J.I. (2019). Slot-Loaded Microstrip Patch Sensor Antenna for high-sensitivity permittivity characterization. Electronics.

[22] Balanis C.A. (2016). Antenna Theory: Analysis and Design.

[23] Memon M.U., Lim S. (2018). Microfluidic high-Q circular substrate-integrated waveguide (SIW) cavity for radio frequency (RF) chemical liquid sensing. Sensors.

[24] Ansari M.A.H., Jha A.K., Akhtar M.J. (2015). Design and application of the CSRR-based planar sensor for noninvasive measurement of complex permittivity. IEEE Sens. J..

[25] Li Q.L., Cheung S.W., Wu D., Yuk T.I. (2016). Optically transparent dual-band MIMO antenna using micro-metal mesh conductive film for WLAN system. IEEE Ant. Wirel. Propag. Lett..

[26] Mestdagh S., De Raedt W., Vandenbosch G.A. (2004). CPW-fed stacked microstrip antennas. IEEE Trans. Ant. Propag..

[27] Saadat-Safa M., Nayyeri V., Khanjarian M., Soleimani M., Ramahi O.M. (2019). A CSRR-based sensor for full characterization of magneto-dielectric materials. IEEE Trans. Microw. Theory Tech..

[28] Kitić G., Crnojević-Bengin V. (2013). A sensor for the measurement of the moisture of undisturbed soil samples. Sensors.

[29] Zhou L., Yu D., Wang Z., Wang X. (2019). Soil water content estimation using high-frequency ground penetrating radar. Water.

[30] Topp G.C., Davis J.L., Annan A.P. (1980). Electromagnetic determination of soil water content: Measurements in coaxial transmission lines. Water Res. Res..

[31] Then Y.L., You K.Y., Dimon M.N., Lee C.Y. (2016). A modified microstrip ring resonator sensor with lumped element modeling for soil moisture and dielectric predictions measurement. Measurement.

[32] Fratticcioli E., Dionigi M., Sorrentino R. (2004). A simple and low-cost measurement system for the complex permittivity characterization of materials. IEEE Trans. Instrum. Meas..

